# Effect of 3-Nitrooxypropanol Combined with Different Feed Additives on Growth Performance, Carcass Traits, Enteric Methane Emissions, and Physiological Responses in Feedlot Beef Cattle Fed a High-Concentrate Finishing Diet

**DOI:** 10.3390/ani14233488

**Published:** 2024-12-03

**Authors:** William Luiz de Souza, Maria Betânia Niehues, Abmael da Silva Cardoso, Victor Valério de Carvalho, Alexandre Perdigão, Tiago Sabella Acedo, Diogo Fleury Azevedo Costa, Luis Fernando Monteiro Tamassia, Maik Kindermann, Ricardo Andrade Reis

**Affiliations:** 1 Department of Animal Science, Faculty of Agricultural and Veterinary Sciences, São Paulo State University, Jaboticabal 14884-900, SP, Brazil; ricardo.reis@unesp.br; 2 Institute for Future Farming Systems, Central Queensland University, Rockhampton, QLD 4701, Australia; d.costa@cqu.edu.au; 3 School of Veterinary Medicine and Animal Science, São Paulo State University, Botucatu 18618-681, SP, Brazil; maria.niehues@unesp.br; 4 Department of Plant and Agroecosystems Science, University of Wisconsin, Madison, WI 53706, USA; abmael2@gmail.com; 5 DSM Nutritional Products, Innovation & Applied Science, São Paulo 01452-001, SP, Brazil; victor.carvalho@dsm-firmenich.com (V.V.d.C.); alexandre.perdigao@dsm-firmenich.com (A.P.); tiago.acedo@dsm-firmenich.com (T.S.A.); 6 DSM Nutritional Products, Aargau, 4303 Kaiseraugst, Switzerland; luis.tamassia@dsm-firmenich.com (L.F.M.T.); maik.kindermann@dsm-firmenich.com (M.K.)

**Keywords:** 3-nitrooxypropanol, beef nutrition, beef production, *Bos indicus*, carbon footprint, greenhouse gas, intensive systems, sustainable intensification, technology adoption

## Abstract

The objective of this study was to evaluate the effect of 3-nitrooxypropanol (3-NOP) in combination with different feed additives on growth performance, carcass traits, meat quality, enteric methane (CH_4_) emissions, nutrient intake and digestibility, and blood parameters in feedlot beef cattle. In experiment (Exp.) 1, one hundred sixty-eight Nellore bulls (initial bodyweight (BW) 410 ± 8 kg) were allocated to 24 pens in a completely randomized block design. In Exp. 2, thirty Nellore bulls (initial BW 410 ± 3 kg) were allocated to a collective pen as one group, and treatments were allocated in a completely randomized design. Three treatments were applied: Control (CTL): monensin sodium (26 mg/kg of dry matter, DM); M3NOP: CTL with 3-NOP (100 mg/kg DM); and Combo: 3-NOP (100 mg/kg DM) with essential oils (100 mg/kg DM), 25-Hydroxy-Vitamin-D3 (0.10 mg/kg DM), organic chromium (4 mg/kg DM), and zinc (60 mg/kg DM). In Exp. 1, bulls in the Combo group had greater (*p* < 0.01) dry matter intake (DMI) at d 28 compared to the CTL and M3NOP groups. During d 0 to 102, bulls’ final BW and average daily gain (ADG) were greater (*p* ≤ 0.03) for the Combo group compared to the CTL. The bulls in the Combo and M3NOP groups had better (*p* < 0.01) feed conversion (FC) and feed efficiency (FE) compared to the CTL. Hot carcass weight (HCW), carcass ADG, and carcass yield were greater (*p* ≤ 0.05) for bulls in the Combo group compared to the CTL and M3NOP groups. The bulls in the Combo group had greater (*p* = 0.01) dressing compared to the M3NOP group. Combo bulls had better (*p* = 0.02) biological efficiency compared to the CTL. The bulls in the Combo group had lower (*p* < 0.01) carcass pH compared to the CTL and M3NOP groups. In Exp. 2, bulls in the Combo group had greater (*p* = 0.04) DMI at d 28 compared to the CTL and had greater (*p* < 0.01) DMI at d 102 compared to the CTL and M3NOP groups. The bulls in the Combo group had greater (*p* = 0.04) HCW compared to the CTL and M3NOP groups and carcass ADG was greater (*p* = 0.04) for bulls in the Combo group compared to the M3NOP group. The bulls in the Combo and M3NOP groups had lower (*p* < 0.01) CH_4_ production (38.8%, g/d), yield (41.1%, g/kg DMI), and intensity (40.8%, g/kg carcass ADG) and higher (*p* < 0.01) H_2_ emissions (291%, g/d) compared to the CTL. Combo bulls had lower (*p* < 0.01) blood glucose and insulin, and higher nutrient intake and digestibility (*p* ≤ 0.05) compared to the CTL and M3NOP groups. Combining 3-NOP with different feed additives improved FC and FE, and reduced enteric CH_4_ emissions. Combo treatment improved growth performance, carcass traits, nutrient intake, and digestibility, and improved glucose and insulin responses in feedlot beef cattle on a high-concentrate finishing diet.

## Simple Summary

The beef industry faces significant challenges in increasing red protein production given the growing population, with projections to be approximately 28% higher by 2050. Additionally, there are increasing government and public pressures for all industries to mitigate greenhouse gases, which include methane. The livestock industry contributes approximately 14.5% of greenhouse gas emissions attributed to human activities. This study investigated the combination of the methane inhibitor 3-nitrooxypropanol with monensin sodium or a feed additive package consisting of essential oils, 25-Hydroxy-Vitamin-D3, an active metabolite of vitamin D3, and carbo-amino-phospho-chelated minerals such as chromium and zinc. The results showed that 3-nitrooxypropanol, regardless of the combination used, reduced daily enteric methane emissions by over 38%, in addition to increasing feed conversion by over 6% when combined with monensin sodium compared to the treatment without 3-nitrooxypropanol. Furthermore, the combination of 3-nitrooxypropanol with the additive package showed better conditions in growth performance, carcass traits, meat quality, blood parameters, and nutrient intake and digestibility. This offers the meat industry more nutritional strategies to meet the growing demand for protein while reducing environmental impacts.

## 1. Introduction

Nowadays, the beef industry faces major challenges to increase productivity and improve product—carcass and meat—quality efficiently and sustainably to meet growing global demand [1,2]. A projection made by the Food and Agriculture Organization of the United Nations [3] estimates a population growth of approximately 28% and a total demand for meat of approximately 75% higher by 2050, in millions of tons. Of this total, 25% is comprised of beef [4]. Alongside the growing demand for meat, concerns related to greenhouse gas emissions arise [5]. The livestock industry contributes approximately 14.5% of greenhouse gas emissions from human activities, with 40% of these emissions coming from methane (CH_4_) originating from ruminants [6], of which 90% comes from ruminal fermentation [7].

Towards more sustainable production, some management and nutritional strategies should be implemented to increase the productivity of beef cattle throughout their life. Examples of nutritional strategies include the use of conserved forage and concentrate supplementation, amongst others, which minimize the effects of seasonal fluctuations in forage production inherent to the climate to assure the continuous supply of nutrients to beef cattle [8,9]. Another alternative is to finish beef cattle in feedlots during periods of low forage availability to establish continuous growth performance and, consequently, increase carcass gains [2]; moreover, this approach promotes the reduction in CH_4_ emissions by yield and intensity, along with the reduction in age at slaughter. In this sense, it has been observed that pathways, including enteric CH_4_ production, can reduce efficiency due to energy loss during their formation [10,11], causing a reduction of up to 12% in the intake of gross energy, and potentially reducing feed efficiency [12].

To mitigate energy losses in addition to management techniques and promote continuous growth in beef cattle throughout their productive life, it is also possible to use feed additives with specific characteristics to reduce enteric CH_4_ emissions, such as 3-nitrooxypropanol (3-NOP), which directly inhibits methanogenesis in beef cattle [13,14]. Its mode of action involves the binding of the molecule to the enzyme methyl-coenzyme M reductase, thus inhibiting the formation of enteric CH_4_, with no negative impact on non-methanogenic bacteria [15]. Reductions in enteric CH_4_ emissions with the inclusion of 3-NOP in the diet of finished cattle can reach up to 90% [16]. However, the need to increase productivity is growing due to the extra cost of the feed additive [17,18]. Therefore, the combination of 3-NOP with other feed additives, like essential oil blends (thymol, eugenol, limonene, and vanillin) [19], 25-Hydroxy-Vitamin-D3, an active metabolite of vitamin D3 [20], and minerals like carbo-amino-phospho-chelated chromium and zinc [21,22], may be beneficial to improve performance and carcass traits. In addition, some minerals, such as chelated chromium and zinc, promote beneficial changes in blood parameters [23,24]; moreover, essential oil [19,25] and 3-NOP, in some cases [26,27], promote an increase in the digestibility of nutrients present in the diet. Given the various roles of feed additives, e.g., antimicrobial properties and rumen fermentation modulation, which affect the metabolism of beef cattle, the combination with 3-NOP was considered as it presents the potential to have beneficial effects, such as improved growth performance through effects on intake, digestibility, and blood parameters. Furthermore, the combination could reduce the carbon footprint per unit of dry matter consumed and per unit of carcass produced, as well as mitigate the daily production of enteric CH_4_ emissions.

We hypothesized that 3-NOP would promote reductions in enteric CH_4_ emissions regardless of the combination, in addition to the fact that the combination of 3-NOP with different additives would result in increases in growth performance based on feed conversion and feed efficiency, including increases in carcass gains and yields and in nutrient intake and digestibility in the high-concentrate finishing diet. The objective of the current work was to evaluate the effect of 3-NOP in combination with different feed additives on growth performance, carcass traits, meat quality, enteric CH_4_ emissions, nutrient intake and digestibility, and blood parameters in feedlot beef cattle on a high-concentrate finishing diet.

## 2. Materials and Methods

The experimental methods were approved by the Ethics Committee on Animal Use of DSM Nutritional Products SA (protocol numbers BR 220121 and BR 211215), following the guidelines of the Animal Research Ethics Committee of São Paulo State University. The experiments were conducted at the Center for Innovation and Applied Science in Ruminants of DSM Nutritional Products, located in Rio Brilhante, Mato Grosso do Sul, Brazil.

### 2.1. Animals, Experimental Design, and Treatments (Exps. 1 and 2)

In Exp. 1, one hundred and sixty-eight Nellore bulls (*Bos indicus*), 16 ± 1 months old with an initial bodyweight (BW) of 410 ± 8 kg, were randomly allocated to 24 pens, using a block design based on the initial bodyweight after 16 h of fasting. There were three treatments conducted with 8 replicates (pens), and 7 bulls in each pen. Each pen had an area of 120 m^2^ (17 m^2^/bull), drinkers with high-flow floats (30 linear cm/bull), and collective feeders (70 linear cm/bull). In Exp. 2, thirty Nellore bulls, 16 ± 1 months old with an initial BW of 410 ± 3 kg, were randomly distributed in a 480 m^2^ group pen (16 m^2^/bull). A completely randomized design was used, with 10 replicates in each treatment (the bulls were considered the experimental unit). The collective pen was equipped with a high-flow float drinker (30 cm linear/bull), 6 electronic feeders (Sistema Intergado^®^, Betim, Minas Gerais, Brazil), and 2 electronic feeders per treatment.

The treatments were the same for Exps. 1 and 2: Control (CTL): monensin sodium (Rumensin 200^®^; main composition: monensin sodium 20%, microtracer 0.06%, and excipients q.s.p. 100%; Elanco Ltd., São Paulo, Brazil) at 26 mg/kg DM; M3NOP: monensin sodium at 26 mg/kg DM and 3-NOP (Bovaer^®^; main composition: Synthetic 3-NOP at a minimum concentration of 10% on a carrier of silicate and dried propylene glycol [16,28]; DSM-Firmenich Nutritional Products Ltd., Kaiseraugst, Switzerland) at 100 mg/kg DM; and Combo: 3-NOP at 100 mg/kg DM; essential oils (Crina^®^ Ruminants; main composition: in 1 g of product—natural or nature-identical aromatic substances (195 mg), including thymol (25–35%), guaiacol (10–15%), eugenol (5–10%), vanillin (10–20%), salicylaldehyde (5–10%), and limonene (20–35%); silicon dioxide (130 mg); lecithins (8 mg); Butylated hydroxytoluene (5 mg); organic carriers (662 mg: palm oil, carob flour, starch, monopropylene glycol, stearic acid, and calcium sulfate) [29,30]; DSM-Firmenich Nutritional Products Ltd., São Paulo, Brazil) at 100 mg/kg DM; 25-Hydroxy-Vitamin-D3 (Hy-D^®^; main composition: 25-Hydroxy-vitamin-D3 (103–105%), water (4%), other sterols (0.9–1.7%), and erythrosine (<0.08 mg/kg) [31]; DSM-Firmenich Nutritional Products Ltd., São Paulo, Brazil) at 0.10 mg/kg DM (corresponding to 4000 IU vitamin D3/kg DM); carbo-amino-phospho-chelated chromium at 4 mg/kg DM (main composition: chromium (5%) and excipients q.s.p. (100%); Tortuga minerals^®^, DSM-Firmenich Nutritional Products Ltd., São Paulo, Brazil); and carbo-amino-phospho-chelated zinc at 60 mg/kg DM (main composition: zinc (24%) and excipients q.s.p. (100%); Tortuga minerals^®^, DSM-Firmenich Nutritional Products Ltd., São Paulo, Brazil).

### 2.2. Management, Animal Feeding, and Diet Analyses (Exps. 1 and 2)

The animal reception program included weighing, deworming, and vaccination following the annual prophylactic schedule. Diets were formulated using the RLM system (Ração de Lucro Máximo, version 3.2, ESALQ, São Paulo, Brazil) (Table 1).

The adaptation to the finishing diet involved a 14-day protocol with two diets offered over seven days each. Upon starting the adaptation and finishing diets, the bulls were fed daily at 8:00 a.m. Diet and residues were collected twice per week in both experiments. These samples were subsequently dried in a forced air circulation oven regulated at 55 °C for 72 h. Next, the samples were ground using a sieve of 1 mm and then subsequent chemical analysis was carried out. The DM (AOAC 934.01), organic matter (OM; calculated: DM—mineral matter (MM)), ether extract (EE; AOAC 920.39), MM (AOAC 942.05), and nitrogen content (AOAC official method 954.01) were converted to crude protein (CP) using a factor of 6.25, as proposed by AOAC [33]. Neutral detergent fiber (NDF) and acid detergent fiber (ADF) were determined with the methodology of Van Soest et al. [34], using a fiber analyzer (Ankom Technologies, New York, NY, USA). Starch was determined using the method described by Hall [35]. The total digestible nutrients (TDNs) of the dietary components were calculated according to the table values and the digestible energy (DE) using the equations proposed by the NRC [36]. The metabolizable energy (ME) was calculated with NASEM [37].

### 2.3. Growth Performance and Carcass Traits (Exps. 1 and 2)

In Exp. 1, the DMI was determined by calculating the difference between the amount of the diet offered and the leftovers collected daily from each pen on the following day. This value was then divided by bulls in the pen. The bulls were under ad libitum intake conditions, with a 3% residue allowance. In Exp. 2, DMI was measured individually for each bull using the electronic feeder system with scales (System Intergado^®^, Intergado Ltd., Betim, Minas Gerais, Brazil) in addition to the sum of individual pellet DMI for each bull from the GreenFeed unit system (C-lock Inc., Rapid City, SD, USA). The diets were provided ad libitum and daily adjustments were made based on the amount of feed refused, with a 5% leftover allowance.

Nellore bulls were weighed individually after a 16 h fasting period on d 0, 28, and 102 of the Exps. 1 and 2. Average daily gain (ADG) was computed using data acquired from weight assessments. Furthermore, calculations were conducted for feed conversion (FC; ratio of DMI/ADG, kg/kg) and feed efficiency (FE; ratio of ADG/DMI, kg/kg).

At the end of Exps. 1 and 2 on d 102, a certified technician collected and analyzed ultrasound images using the BIA/DGT Brazil software (https://www.dgtbrasil.com.br/software-bia-bovinos/). Measurements of *Longissimus* muscle (LM) area, rib fat thickness (RFT), marbling, and rump fat thickness were obtained via ultrasound (ALOKA SSD 100 VET^®^, Aloka, Tokyo, Japan) utilizing a 17 cm linear probe operating at a frequency of 3.5 MHz. After measuring the carcass traits with ultrasound, the bulls were transported to a slaughterhouse (Campo Grande, Mato Grosso do Sul, Brazil) located 165 km from the experiment site. The slaughter procedures adhered to the standards established by the slaughterhouse following prevailing regulations governing the slaughter of cattle. The hot carcass weight (HCW) at the time of slaughter was determined. The initial HCW was established based on a yield derived from the reference slaughter conducted at the beginning of the Exps. 1 and 2, resulting in the following equation: initial HCW = initial BW × 0.5226 for further calculations. The carcass ADG in kg/day was calculated as follows: (final HCW − initial HCW)/102 days. The dressing % was calculated as follows: (final HCW/final BW) × 100. The carcass yield percentage was determined as follows: ((final HCW − initial HCW)/(final BW − initial BW)) × 100. The biological efficiency (kg DM/kg HCW) was calculated as follows: (total DMI intake over 102 days)/((final HCW − initial HCW)).

### 2.4. Meat Quality (Exp. 1)

Two bulls were selected per pen for meat quality assessment, totaling sixteen per treatment. Carcass pH was measured 24 h after slaughter in a cold room at 4 °C, in the region of the LM (12th rib), using a pH meter (model HI 99163, Hanna Instruments, São Paulo, Brazil). Samples for quality assessments were collected from the LM between the 11th and 13th rib, stored in vacuum-sealed bags, and frozen. Meat color analyses (L*, a*, and b*) were performed at 24 and 72 h. The steaks were thawed in a refrigerator at 6 °C for a duration of 24 h and exposed to oxygen for 30 min. Measurements at four different points on the samples were conducted using a portable spectrophotometer (model CM2500d, Konica Minolta, São Paulo, Brazil) with D65 illumination and a 10° standard observer angle, calibrated with black and white standards [38].

In the analyses of shear force and cooking losses, 3 steaks (2.54 cm thick) were selected and thawed under refrigeration at 6 °C for a duration of 24 h, with the initial weight of the sample being determined. A thermometer was used at the geometric center of each steak, and the steaks were heated in an industrial electric oven (model F130/L, Flecha de Ouro Ind. e Com. Ltd., São Paulo, Brazil) set to a target temperature of 170 °C until they reached an internal temperature of 40 °C. After turning the steaks, they were cooked until the internal temperature reached 71 °C. The steaks were then cooled to room temperature, and the difference between the final and initial weights of each steak was calculated to determine the cooking loss. The steaks were subsequently stored in refrigeration at 6 °C for a duration of 24 h and later analyzed for shear force. The analysis was performed using five cylinders with a diameter of 1.27 cm to remove fragments of the steaks along the muscle fiber direction and analyzed using a texturometer (TMS-PRO, Food Technology Corporation, Sterling, VA, USA). For calculation purposes, the values of the five cylinders were used to obtain the shear force value expressed in Newtons (N). All procedures for these analyses were described by the AMSA [39].

Myoglobin determination was performed using a steak (1.27 cm thick), in which the connective tissue and external fat were removed, and the meat was reduced to cubes and quickly frozen in liquid nitrogen. Myoglobin extraction and quantification were performed following the methodology established by Warris [40] and adapted by Hunt et al. [41]. The concentration of myoglobin extracted was quantified by measuring the absorbance at 433 nm. The calculations were performed using a molar extinction coefficient of 1.14 × 10^5^ M/cm, the molecular weight of myoglobin, and the corresponding dilution factor. Total lipids were determined in the meat samples in triplicate, following the methodology of Bligh and Dyer [42]. The methodology of Sorensen and Jorgensen [43] was used for the analysis of lipid oxidation. Five grams of meat from each sample were used, in triplicate, and homogenized for 1 min with 15 mL of trichloroacetic acid, with subsequent absorbance readings at 530 and 632 nm. A standard curve containing 5 points was created using the tetraethoxypropane solution of known concentration, thus obtaining the malonaldehyde (MDA) concentrations through the standard curve equation, expressed in mg MDA/kg of meat.

### 2.5. Blood Variables (Exp. 2)

Blood samples were collected from thirty bulls on d 0, 28, and 102 of the feedlot period. The samples were obtained via venipuncture of the jugular vein using appropriate tubes (BD Vacutainer^®^, São Paulo, Brazil), collecting approximately 10 mL of blood per sample. Following collection, the blood samples were immediately transferred to ice and subsequently centrifuged at 3000 rpm for 15 min at 4 °C. After, serum was promptly transferred to a polypropylene container and stored at −20 °C. Biochemical markers assessed in the blood included glucose, urea, albumin, creatinine, total proteins, cholesterol, triglycerides, aspartate aminotransferase (AST), and gamma-glutamyl transferase (GGT). These markers were quantified using commercial Bioclin^®^ kits (Bioclin^®^, Belo Horizonte, Minas Gerais, Brazil) and absorbance measurements were conducted with a spectrophotometer (SBA 200, CELM, São Caetano do Sul, Brazil), according to the manufacturer’s instructions. Serum levels of insulin and Insulin-like Growth Factor 1 (IGF-1) were measured using the Immulite 1000^®^ commercial kit (Diagnostic Products Corporation, Los Angeles, CA, USA).

### 2.6. Digestibility of Nutrients (Exp. 2)

Diet and fecal collections were conducted at three specific time points on d 28, 56, and 84 of the feedlot periods, representing the start, middle, and end of the finishing phase. These collections were conducted over five days to evaluate the digestibility of DM, organic matter (OM), CP, EE, NDF, ADF, and starch. Diet samples and leftovers were collected once daily. These samples were identified and stored at −20 °C. Fecal samples were collected three times daily (08:00 a.m., 01:00, and 05:00 p.m.) directly from the ground in the pens from thirty bulls, immediately after the bulls defecated. Fecal samples were placed on ice and stored at −20 °C shortly after collection. Diet, leftover, and fecal samples were dried at 55 °C for a duration of 48 h in a forced-air oven and ground using a Wiley mill (Model 4 Wiley, Thomas Scientific, Swedesboro, NJ, USA) to pass through both 2 mm and 1 mm sieves. After grinding, the samples were sent for composition analysis, where indigestible neutral detergent fiber (iNDF) was used as an internal marker [44,45]. Apparent nutrient digestibility was then calculated using the following formula: nutrient digestibility (%) = 100 − (100 × [nutrient content in feces × iNDF content in the feed]/[nutrient content in feces × iNDF content in feces]).

### 2.7. Greenhouse Gas Emissions (Exp. 2)

Measurement data for enteric CH_4_ and hydrogen (H) gas emissions were collected from thirty bulls individually during the total feedlot period of 102 days for 24 h. Enteric CH_4_ emissions were determined using the GreenFeed unit (C-lock Inc., Rapid City, SD, USA), following the methods described by Della Rosa et al. [46]. Through this system, it was possible to measure the emissions of gases from individual bulls. The bulls voluntarily approached the equipment and received a small amount of additive-free pelleted feed with the composition of 88.5% DM, 17.0% CP, 3.00% EE, 1.20% calcium, and 0.60% phosphorus. Average daily emissions were estimated by aggregating data from several visits throughout the observation period. The equipment was programmed to produce 6 drops of concentrate pellets per visit and with a minimum interval of 4 h between each visit. For each feeding period, a maximum of five drops of feed containing an average of 35.5 g of pellets was allowed, with an interval of 35 s between drops. During all measurements, the airflow within the system was greater than 40 L/s. After the measurements, daily enteric CH_4_ emissions were calculated in g/d and expressed as yield based on DMI (g CH_4_/kg DMI), as intensity based on carcass ADG (g CH_4_/kg carcass ADG), and as enteric H_2_ emissions in g/d.

### 2.8. Statistical Analysis (Exps. 1 and 2)

In both Exps., statistical analyses were conducted using SAS software version 9.4 (SAS Institute, Cary, NC, USA). The Cramér–von Mises normality test was applied to all data, followed by ANOVA, and the Tukey test was employed for mean comparisons. In Exp. 1, the experimental units were the pens (*n* = 24; 7 bulls/pen). The experimental design was randomized blocks, using the initial fasting BW of the bulls for block allocation. The treatments were as follows: Control (CTL, *n* = 56 bulls), M3NOP (*n* = 56 bulls), and Combo (*n* = 56 bulls). The analysis included data on growth performance, carcass traits, and meat quality. In Exp. 2, a completely randomized design was used, where individual bulls in the CTL (*n* = 10 bulls), M3NOP (*n* = 10 bulls), and Combo (*n* = 10 bulls) treatments were considered as experimental units, and treatments were considered as fixed effects. Conducting Exp. 2 allowed us to assess the reproducibility of the results at the individual bull level, a crucial factor in bull model research to ensure that the findings are robust and replicable. The analysis included data on growth performance, carcass traits, greenhouse gas measurements, blood parameters, and nutrient intake and digestibility. Blood parameters, nutrient intake, and digestibility data were analyzed as repeated measurements. Due to potential inter-bull variability, blood parameter measurements on d 0 were tested and, when significant, used as covariates for measurements on d 28 and d 102 to mitigate the impact of inter-bull variability on the analysis and allow a more accurate assessment of treatment effects on repeated measurements. The Bayesian Information Criterion was employed to select the best covariance structure. For all statistical results, a significance level of *p* ≤ 0.05 was considered.

## 3. Results

### 3.1. Growth Performance, Carcass Traits, and Meat Quality (Exp. 1)

The effects of the CTL, M3NOP, and Combo treatments were detected (*p* ≤ 0.05) on growth performance, carcass traits, and meat quality (Table 2). The Combo treatment had a DMI value 5.56% (*p* < 0.01) higher than the CTL and M3NOP treatments on d 28. On d 102, BW and ADG were higher by 13 kg (*p* = 0.03) and 0.140 kg/d (*p* = 0.01), respectively, for the Combo treatment compared to the CTL, with no differences found for the M3NOP treatment. The Combo and M3NOP treatments showed an improvement in FC of 5.87% (*p* < 0.01) and an FE of 5.58% (*p* < 0.01) compared to the CTL. No differences were detected (*p* ≥ 0.07) among treatments for initial BW, BW at d 28, ADG at d 28, FC at d 28, FE at d 28, and DMI at d 102 (Table 2).

The Combo treatment had 13 kg in final HCW (*p* < 0.01), 0.113 kg/d in carcass ADG (*p* < 0.01), and 3.42% in carcass yield (*p* = 0.05) higher than the CTL and M3NOP treatments. In carcass traits, the Combo treatment resulted in a dressing percentage 1.75% higher (*p* = 0.01) than the M3NOP treatment, with no difference found in the CTL. Biological efficiency showed a lower (*p* = 0.02) requirement for DMI to convert 1 kg of HCW, which was approximately 7.69% less in the Combo treatment compared to the CTL treatment, with no difference found in the M3NOP treatment. No differences (*p* ≥ 0.21) were observed between treatments for initial HCW, LM area, marbling, 12th-rib fat thickness, and rump fat thickness (Table 2). The Combo treatment reduced the carcass pH value by 1.52% (*p* < 0.01) when compared to the CTL and M3NOP treatments. No difference (*p* ≥ 0.17) between treatments was found for shear force, cooking loss, meat color (Chroma) at 24 and 72 h, myoglobin, total lipids, and lipid oxidation (Table 2).

### 3.2. Growth Performance and Carcass Traits (Exp. 2)

The effects of the CTL, M3NOP, and Combo treatments were detected (*p* ≤ 0.04) on growth performance and carcass traits (Table 3). Over d 28, bulls in the Combo treatment had DMI values 5.51% (*p* = 0.04) higher than the CTL treatment, with no difference found in the M3NOP treatment. However, on d 102, bulls in the Combo treatment had DMI values 8.20% (*p* < 0.01) higher than the CTL and M3NOP treatments. No differences (*p* ≥ 0.19) between treatments were observed for initial BW, BW at d 28, ADG at d 28, FC at d 28, FE at d 28, BW at d 102, ADG at d 102, FC at d 102, and FE at d 102 (Table 3).

The Combo treatment had final HCW 14 kg (*p* < 0.01) and carcass ADG 0.118 kg/d (*p* < 0.01) higher than the CTL treatment and no differences compared to the M3NOP treatment. No differences were observed (*p* ≥ 0.11) between treatments for initial BW, carcass yield, biological efficiency, LM area, marbling, 12th-rib fat thickness, and rump fat thickness (Table 3).

### 3.3. Greenhouse Gas Measurements

An effect (*p* < 0.01) was detected between treatments for greenhouse gas measurements (Table 4). The M3NOP and Combo treatments resulted, on average, in reductions of 38.8% in daily CH_4_ production (*p* < 0.01), 41.1% in CH_4_ yield (*p* < 0.01), and 40.8% in CH_4_ intensity (*p* < 0.01) compared to the CTL treatment. In addition, the Combo and M3NOP treatments had higher (*p* < 0.01) H_2_ emissions by 291% more than the CTL treatment. No differences (*p* ≥ 0.44) were found between the treatments based on the total drops per day, amount in kilograms, and time in minutes per day (Table 4).

### 3.4. Blood Parameters (Exp. 2)

The effects on blood parameters were observed, which were significantly influenced by treatments (*p* ≤ 0.05) and by the treatment × time interaction (*p* ≤ 0.01) (Table 5). Serum insulin and glucose levels were affected by the treatment × time interaction (*p* ≤ 0.01), with all treatments showing increased levels on d 28 and 102. The Combo treatment exhibited reduced serum insulin and glucose levels compared to the average levels observed in the CTL and M3NOP treatments. A treatment × time interaction (*p* = 0.01) was also noted for serum albumin, with lower concentrations on d 102 for the Combo treatment compared to the levels of the CTL and M3NOP treatments. The Combo treatment showed a reduction in AST by 9.04% (*p* = 0.04) and cholesterol by 12.4% (*p* < 0.01) compared to the CTL and M3NOP treatments. No differences were observed for treatment (*p* ≥ 0.09) or treatment × time interaction (*p* ≥ 0.17) for variables like IGF-1, total protein, creatinine, GGT, and triglycerides.

### 3.5. Intake and Digestibility of Nutrients

The effects of treatments were detected (*p* ≤ 0.05) on nutrient intake, with the Combo treatment having higher intakes of DMI, OM, CP, ADF, NDF, and starch by 7.95%, 8.08%, 7.68%, 8.04%, and 7.27%, respectively, compared to the CTL and M3NOP treatments, with the exception of EE (*p* = 0.18). No difference was detected (*p* ≥ 0.85) for the interaction treatment x time on nutrient intake, including DM, OM, CP, EE, ADF, NDF, and starch (Table 6).

The interaction between treatment x time detected an effect (*p* = 0.04) on NDF digestibility, in which the Combo treatment increased from d 0 to d 102 when compared to the CTL and M3NOP treatments, which decreased and maintained the behavior, respectively (Table 6). No difference (*p* ≥ 0.07) was found for the treatment x time interaction on DM, OM, CP, EE, ADF, and starch. However, the treatments had an effect (*p* ≤ 0.05) on nutrient digestibility, in which the Combo treatment had greater digestibility of CP, EE, and starch by 1.23%, 4.25%, and 0.88%, respectively, compared to the CTL and M3NOP treatments. No differences (*p* ≥ 0.08) were observed between the treatments for the digestibility of DM, OM, and ADF (Table 6).

## 4. Discussion

Our hypothesis confirmed that adding 3-NOP, regardless of the combination, would mitigate enteric CH_4_ emissions and that the combination of 3-NOP with different feed additives would result in better growth performance, FC and FE, in addition to promoting carcass gains and yields, with further improvements in the digestibility of nutrients in a high-concentrate finishing diet. The use of CH_4_ inhibitors may lead to high hydrogen in the rumen, hence why it would be important to consider the use of other feed ingredients to ameliorate rumen health and improve overall performance. Recent research evaluated the possibility of combining different additives to promote growth performance and improvements in carcass traits [19,25,47].

The M3NOP and Combo treatments resulted in significant reductions in CH_4_ emissions in terms of absolute production, yield, and intensity, compared to the CTL treatment. Researchers are in agreement that the addition of 3-NOP at different doses and conditions to ruminant feed points to its efficiency in reducing CH_4_ emissions in beef cattle [5,16,27,48] and dairy cows [26,49,50,51]. The latter authors found that 3-NOP is able to reduce enteric CH_4_ emissions in different metrics, like daily production, DMI, energy intake, carcass ADG, or milk production, although not the focus of this research. Mitigation of enteric CH_4_ emissions in beef cattle can reach up to 90% with the use of 3-NOP [16]. However, a global review on the use of 3-NOP by Yu et al. [5] suggests that reductions in enteric CH_4_ emissions tend to approach 30% in daily production in beef cattle, which corroborates our findings of a 38.8% reduction in daily production. Yu et al. [5] also emphasized that oscillations in enteric CH_4_ mitigation depend mainly on the composition of the diet (e.g., % NDF) and the doses of 3-NOP used in the studies. These reductions are attributed to the high molecular specificity of 3-NOP in binding to the enzyme methyl-coenzyme M reductase, thus inhibiting it. This enzyme is responsible for catalyzing the formation of CH_4_ in the last stage of methanogenesis in archaea [15], resulting in the availability of CO_2_ and H_2_ to the ruminal environment. In this study, a 291% increase in enteric H_2_ emissions was found. These findings are complemented by other literature, which also observed reductions in enteric CH_4_ emissions and an increase in H_2_ emissions [5,26]. This rise in CO_2_ and H_2_ is expected because in methanogenesis they both act as substrates, where CO_2_ is reduced by H_2_ to form CH_4_ by methanogenic archaea. However, the action of 3-NOP in inhibiting the final stage of methanogenesis likely reduces both the methanogenic population and CH_4_ production, leading to an accumulation of H_2_ in the rumen and, consequently, an increase in its enteric emission of H_2_. Zhang et al. [27] highlight the importance of experiments investigating the combinations of 3-NOP with other additives, aiming to identify those that, when implemented together, provide better results; however, our findings did not differ between the combinations of 3-NOP with different strategies (M3NOP and Combo) regarding CH_4_ and H_2_ enteric emissions.

Exps. 1 and 2 are complementary in elucidating the effects of combining 3-NOP with different feed additives. We highlight the improvements in FC of 5.87% and FE of 5.50% in the M3NOP and Combo treatments when compared to the CTL treatment in Exp. 1. This increase in FC and FE may be related to several factors linked to the multiple additives present in the Combo treatment. However, the combination of 3-NOP with monensin sodium in the M3NOP treatment showed better FC and FE compared to the CTL, which may have enhanced the effect observed in the Combo treatment. Vyas et al. [48] reported that the addition of 3-NOP promoted an improvement in feed efficiency of 5% in the growing phase in the feedlot and 3% in the finishing phase of beef cattle. We cannot rule out a possible mechanism of hydrogen utilization that stops participating in the formation of CH_4_, with part being emitted via H_2_ and part as dissolved H ([H]) in the ruminal content. According to Ungerfeld et al. [52], this [H] can be directed to sinks such as the propionate pathway, consequently driving improvements in feed efficiency and beef cattle growth performance. The increase in rumen [H] concentration shows a negative correlation with acetate levels and a positive correlation with propionate levels [53,54]. Although we did not measure dissolved hydrogen ([H]) due to quantification challenges, we acknowledge it is a potential contributor to energy processes like the propionate formation, leaving a gap for future studies focused on its impact on overall fermentation dynamics and the association with changes in microbial populations

As for the higher DMI in the Combo bulls, this occurred due to the inclusion of monensin sodium in the CTL and M3NOP treatments, in accordance with Torres et al. [55] who conducted a meta-analysis with beef cattle, demonstrating that the replacement of monensin sodium with the inclusion of essential oils in the diets increased DMI. Corroborating our findings, a meta-analysis carried out by Duffield et al. [56] reported a 3% reduction in DMI when a dose of monensin sodium of 28.1 mg/kg DM was supplemented in the diet of beef cattle. Gadberry et al. [57] also performed a meta-analysis with data from heifers and beef cows and observed a DMI reduction effect of around 4.30% at daily doses ranging from 125 to 200 mg/d per animal. The literature is in line with Exp. 1, in which, on d 28, we observed a lower DMI of 5.56% on average for the CTL and 3-NOP treatments (both containing monensin sodium) compared to the Combo treatment. In Exp. 2, on d 28, a 6.78% lower DMI was observed for the CTL compared to the Combo treatment and, on d 102, an 8.20% reduction was observed for the average of the CTL and 3-NOP treatments compared to the Combo treatment. These findings in Exps. 1 and 2, despite the alignment observed in the literature on treatments containing monensin sodium in the reduction in DMI, were slightly higher. This increase was probably due to the stimulation of DMI by essential oils or other components contained in the Combo treatment, and the reduction observed with the addition of monensin sodium to the diet of beef cattle. Consequently, higher BW and ADG were observed in the Combo treatment in Exp. 1, due to the metrics of FC, FE, and higher DMI in relation to the other treatments. The literature demonstrates increases in BW and ADG when essential oils [19,25] and chromium [58,59,60,61] are included in beef cattle diets. The complementarity of the results in Exps. 1 and 2 in growth performance was also observed in carcass traits, with the Combo treatment being superior to the others. In the CTL and M3NOP treatments, which contain monensin sodium, no effect was observed in stimulating carcass deposition or improving intake and efficiency characteristics [56]. In contrast, the Combo treatment contained in its composition several elements with proven capacity to increase carcass gains and yields. However, the essential oil (containing thymol, eugenol, limonene, and vanillin) included in the diet of feedlot beef cattle results in carcass traits like those presented by animals that received monensin sodium [25,62], highlighting the essential oil as a viable alternative for use in ruminants. Another component of the Combo treatment is 25-Hydroxy-Vitamin-D3, which, when included at a dose of 1 mg/animal/day in the finishing diet of Nellore cattle, increased yield by 0.96% [63] and increased HCW by 4.2 kg [64]. In addition, the inclusion of 25-Hydroxy-Vitamin-D3 in finishing diets for feedlot beef cattle increased the expression of Insulin-like Growth Factor (IGF)-1, IGF-2, mammalian target of rapamycin (mTOR), and myostatin (MSTN) genes, which are genes related to muscle anabolism, protein turnover, and regulation of muscle growth [65].

One challenge for researchers using complex mixtures is that many may include proprietary formulations. It can be difficult to use analytical techniques like gas or liquid chromatography–mass spectrometry. These methods may not be feasible or suitable, and may involve lengthy sample preparations and separations. Additionally, techniques like colorimetric assays require the development of specific methods for each compound. Batley et al. [66] suggested the use of Fourier-transform infrared-attenuated total reflectance spectroscopy to efficiently identify compounds by generating unique spectra in a faster and more cost-effective manner. Another element present in the Combo treatment that may have contributed to better carcass traits was chromium. When chromium was included in feedlot diets for beef cattle, an increase in HCW was observed [21,22,59,60]. However, there are studies in the literature indicating that the addition of chromium in finishing diets for beef cattle, compared to the Control group, did not show an increase in HCW [24,67,68,69,70]. This same inconsistency is also reported in zinc administration, with some studies showing an increase in carcass traits [71], while others show no effect in relation to the Control treatment [21,22,68]. However, the intentional use of a mixture containing chromium and zinc increases HCW, as observed in our Exps. 1 and 2, with a consequent increase in gain and carcass yield characteristics. In view of this, Budde et al. [21], studying the effect of including chromium and zinc in combination in feedlot cattle diets, compared it with animals that received only zinc and observed an increase of 8.5 kg in HCW. These findings corroborate our results in HCW found in Exps. 1 and 2, where there was an increase of 12 and 14 kg, respectively, in addition to better biological efficiency in Exp. 1 for the Combo treatment compared to the CTL treatment, which demonstrated a lower requirement for DMI to convert 1 kg of HCW. These superior results are probably the effect of the combination of additives that allowed better results in terms of carcass. The Combo treatment improved the carcass pH range 24 h post-mortem, as found by Matthews et al. [72], who reported lower pH values at 24 h for pigs supplemented during finishing with chromium chelated with the amino acid methionine. In addition, there is the possibility that the chromium mechanism acts as a stress-mitigating element, reducing cortisol secretion [73], which could mitigate the stress of transportation to the slaughterhouse, which interferes with carcass pH values [74].

In Exp. 2, all treatments showed an increase in serum glucose and insulin concentrations over time. However, the Combo treatment, as described in the results, showed lower glucose and insulin concentrations compared to the CTL and M3NOP treatments from d 28 to d 102. This lower concentration suggests lower insulin resistance, which may promote greater DMI during the feedlot phase and increased carcass traits such as HCW, a result found and previously discussed in the Combo treatment in Exps. 1 and 2. Therefore, there is evidence that minerals, such as chromium and zinc, which are contained in the Combo treatment, are effective in improving glucose tolerance and reducing insulin resistance in beef cattle [24,75,76,77], increasing insulin binding and the number of insulin receptors and enzymes, and promoting insulin sensitivity through β-cells and facilitating its internalization [78,79], thus positively regulating gluconeogenesis [24]. Insulin reduces gluconeogenesis by suppressing the secretion of glucagon from the pancreas [80].

AST and cholesterol parameters were compared to reference values [81,82] and demonstrated normal concentrations. However, the Combo treatment, as illustrated in the results, presented lower AST and cholesterol concentrations. The literature highlights potential health benefits linked to minerals like chromium and zinc [23,83,84,85], corroborating our findings in Exp. 2 for the Combo treatment. Soumar et al. [23] included chromium and zinc in the diet of lambs before transport and observed that in all treatments there were increases in AST concentration after transport, but the supplemented animals had lower AST concentrations compared to the CTL treatment, indicating an enhancement in the health indicator. This suggests that, possibly, the inclusion of chromium and zinc in the Combo treatment improves the health of bulls in feedlot situations under a high-concentrate diet. The serum albumin concentration in the Combo treatment remained unchanged on d 28 in relation to the initial concentration, while in the CTL and M3NOP treatments, there was an increase. In addition, albumin in the Combo treatment increased on d 102, although the concentrations remained lower than those observed in the CTL and M3NOP treatments, corroborating Hassan et al.’s [84] study that showed lower albumin concentrations in rats supplemented with chromium or zinc in the diet, which is associated with reduced serum AST concentrations. Furthermore, these minerals also influence lipid metabolism, reducing cholesterol levels. Soumar et al. [23] found that the inclusion of chromium and zinc led to a reduction in cholesterol concentrations in animals. Experiments with rats show that minerals like chromium and zinc can promote the reduction in serum levels of AST, albumin, and total cholesterol, in addition to effects on insulin sensitivity and resistance [84,85].

As previously discussed, bulls in the Combo group had higher DMI compared to the CTL and M3NOP groups, which in turn resulted in a higher intake of OM, CP, NDF, ADF, and starch (Table 6). This condition, depending on digestibility, can favor growth performance and, consequently, improve carcass traits. Meschiatti et al. [25] showed that treatment containing essential oils, compared to monensin sodium in feedlot beef cattle, resulted in a higher intake of DM, CP, NDF, and starch. Furthermore, Toseti et al. [19] demonstrated that the inclusion of a combination of essential oils together with alpha amylase in diets of finishing beef cattle increased the intake of DM, OM, CP, NDF, ADF, EE, and starch compared to the inclusion of monensin sodium. Nutrient digestibility was higher in bulls that received the Combo treatment, specifically for CP, EE, NDF, and starch. One possible explanation is the action of the essential oil present in this treatment, which can positively affect nutrient digestibility. Toseti et al. [19] demonstrated greater digestibility of CP and EE in finishing beef cattle. Li et al. [86] reported that the inclusion of essential oil in an in vitro experiment resulted in increased NDF digestibility. The presence of 3-NOP may have enhanced the improved effects on digestibility values observed in the Combo group. Several studies in the literature show positive results of 3-NOP in ruminants, including increased digestibility of CP [10,26,27], NDF [49], and starch [27] in the diet. Although 3-NOP is present in the M3NOP treatment and does not show significant differences compared to the CTL treatment, it may be related to the similarity in DMI between the CTL and M3NOP treatments. Moreover, monensin sodium has the main effect of reducing DMI in beef cattle [56,57].

## 5. Conclusions

The addition of 3-NOP to our experimental diets, both with monensin sodium or with a feed additive package containing essential oil, 25-Hydroxy-Vitamin-D3, and carbo-amino-phospho-chelated minerals such as chromium and zinc, proved to be an effective mitigator of enteric CH_4_ emissions, with an average reduction of 38.8%, promoting sustainability within the beef cattle feedlot operation. Furthermore, including 3-NOP with monensin sodium improved feed conversion and feed efficiency compared to the exclusive inclusion of monensin sodium in high-concentrate diets. The combination of 3-NOP with a feed additive package containing essential oil, 25-Hydroxy-Vitamin-D3, and carbo-amino-phospho-chelated minerals, such as chromium and zinc, positively affected several parameters, such as growth performance and carcass traits. The effects resulted in improved intake and digestibility of nutrients, accompanied by changes in the responses to serum glucose, insulin, albumin, and cholesterol, indicating improved glucose tolerance and reduced insulin resistance. Furthermore, improved aspartate aminotransferase parameters were observed, which is a crucial indicator of liver health in finishing beef cattle under high-concentrate diets.

It is recommended to investigate the effects of 3-NOP across various diets, its interaction with other feed additives, and its impact on health, growth performance, and carcass characteristics at different cattle production stages to optimize efficiency and sustainability in beef production. Future studies should also examine the potential of excess H, redirected from CH_4_ formation, to support energy-yielding pathways like propionate metabolism.

## Figures and Tables

**Table 1:** Ingredients and nutritional profile of diets (dry matter basis) in Exps. 1 and 2.

Item	Diet Adaptation 1			Diet Adaptation 2			Diet Finishing		
	CTL ^1^	M3NOP ^2^	Combo ^3^	CTL	M3NOP	Combo	CTL	M3NOP	Combo
Ingredients, % DM									
Sugarcane bagasse, %	30.0	30.0	30.0	20.0	20.0	20.0	10.0	10.0	10.0
Soybean meal, %	11.0	11.0	11.0	6.35	6.35	6.35	1.70	1.70	1.70
Ground corn, %	50.0	50.0	50.0	59.5	59.5	59.5	69.0	69.0	69.0
Cottonseed, %	5.00	5.00	5.00	10.0	10.0	10.0	15.0	15.0	15.0
Urea, %	1.00	1.00	1.00	1.15	1.15	1.15	1.30	1.30	1.30
Mineral supplement ^4^, %	3.00	3.00	3.00	3.00	3.00	3.00	3.00	3.00	3.00
Monensin sodium, mg/kg DM	26.0	26.0	-	26.0	26.0	-	26.0	26.0	-
Essential oils, mg/kg DM	-	-	100	-	-	100	-	-	100
25-Hydroxy-Vitamin-D3, mg/kg DM	-	-	0.10	-	-	0.10	-	-	0.10
3-nitrooxypropanol, mg/kg DM	-	100	100	-	100	100	-	100	100
Chemical composition (DM-basis)									
Dry matter, %	89.5	89.5	89.5	90.3	90.3	90.3	90.5	90.5	90.5
Organic matter, % DM	84.6	84.6	84.6	85.2	85.2	85.2	85.3	85.3	85.3
Crude protein, % DM	16.2	16.2	16.2	15.4	15.4	15.4	14.8	14.8	14.8
Mineral matter, % DM	4.93	4.93	4.93	5.05	5.05	5.05	5.24	5.24	5.24
Total digestible nutrients, % DM	69.8	69.8	69.8	74.2	74.2	74.2	81.1	81.1	81.1
Neutral detergent fiber, % DM	32.2	32.2	32.2	27.8	27.8	27.8	26.5	26.5	26.5
Acid detergent fiber, % DM	22.8	22.8	22.8	19.3	19.3	19.3	18.4	18.4	18.4
PeNDF ^5^, % DM	27.2	27.2	27.2	22.2	22.2	22.2	17.2	17.2	17.2
Starch, % DM	35.5	35.5	35.5	42.2	42.2	42.2	49.0	49.0	49.0
Vitamin D3, IU/kg DM	510	510	4510	510	510	4510	510	510	4510
Chromium, mg/kg DM	0.20	0.20	0.60	0.20	0.20	0.60	0.20	0.20	0.60
Zinc, mg/kg DM	60.0	60.0	120	60.0	60.0	120	60.0	60.0	120
ME, Mcal/kg DM	2.52	2.52	2.52	2.68	2.68	2.68	2.93	2.93	2.93

Abbreviations: DM—dry matter; ME—metabolizable energy; and Mcal—megacalorie. ^1^ CTL: monensin sodium (Rumensin 200^®^; Elanco Ltd., São Paulo, Brazil) at 26 mg/kg DM. ^2^ M3NOP: monensin sodium at 26 mg/kg DM and 3-nitrooxypropanol (Bovaer^®^; DSM-Firmenich Nutritional Products Ltd., Kaiseraugst, Switzerland) at 100 mg/kg DM. ^3^ Combo: 3-nitrooxypropanol at 100 mg/kg DM; essential oils (Crina^®^ Ruminants; DSM-Firmenich Nutritional Products Ltd., São Paulo, Brazil) at 100 mg/kg DM; 25-Hydroxy-Vitamin-D3 (Hy-D^®^; DSM-Firmenich Nutritional Products Ltd., São Paulo, Brazil) at 0.10 mg/kg DM (corresponding to 4000 IU vitamin D3/kg DM); carbo-amino-phospho-chelated chromium at 4 mg/kg DM (Tortuga minerals^®^; DSM-Firmenich Nutritional Products Ltd., São Paulo, Brazil); and carbo-amino-phospho-chelated zinc at 60 mg/kg DM (Tortuga minerals^®^; DSM-Firmenich Nutritional Products Ltd., São Paulo, Brazil). ^4^ Mineral and vitamin supplement containing (per kg DM): 150 g calcium; 16 g phosphorus; 36 g sulfur; 20 g magnesium; 34 g potassium; 56 g sodium; 8 mg cobalt; 540 mg copper; 6.7 mg chromium; 27.5 mg iodine; 1070 mg manganese; 6.70 mg selenium; 2000 mg zinc; 168,000 IU vitamin A; 17,000 IU vitamin D3; 1740 IU vitamin E; 90 mg biotin; 1.35 × 10^11^ CFU *Saccharomyces cerevisiae*. Manufactured by DSM Nutritional Products, São Paulo, Brazil. ^5^ peNDF—physically effective neutral detergent fiber (Lammers et al. [32]); the peNDF in the diet, based on DM, was determined by the fraction kept on the 1.17 mm sieve.

**Table 2:** The effect of combining 3-nitrooxypropanol with different feed additives on growth performance, carcass traits, and meat quality of feedlot Nellore bulls fed a high-concentrate finishing diet in Exp. 1.

Item	CTL ^1^	M3NOP ^2^	Combo ^3^	SEM	*p*-Value
Growth performance					
Initial bodyweight, kg	410	410	410	8.17	0.83
d 28 dry matter intake, kg/d	11.2 ^b^	10.9 ^b^	11.7 ^a^	0.22	<0.01
d 28 average daily gain, kg/d	1.816	1.795	1.918	0.06	0.34
d 28 feed conversion, kg/kg	6.23	6.11	6.14	0.21	0.91
d 28 feed efficiency, kg/kg	0.162	0.164	0.163	0.01	0.93
d 28 bodyweight, kg	462	460	464	8.18	0.46
d 102 dry matter intake, kg/d	12.1	11.8	12.4	0.26	0.07
d 102 average daily gain, kg/d	1.733 ^b^	1.808 ^ab^	1.873 ^a^	0.03	0.01
d 102 feed conversion, kg/kg	6.98 ^a^	6.54 ^b^	6.60 ^b^	0.13	<0.01
d 102 feed efficiency, kg/kg	0.144 ^a^	0.154 ^b^	0.152 ^b^	0.01	<0.01
Final bodyweight, kg	588 ^b^	594 ^ab^	601 ^a^	8.63	0.03
Carcass traits					
Initial hot carcass weight, kg	214	213	213	4.47	0.57
Final hot carcass weight, kg	332 ^b^	334 ^b^	345 ^a^	5.29	<0.01
Carcass average daily gain, kg/d	1.159 ^b^	1.188 ^b^	1.287 ^a^	0.02	<0.01
Dressing, %	56.5 ^ab^	56.3 ^b^	57.3 ^a^	0.23	0.01
Carcass yield, %	67.0 ^b^	65.7 ^b^	68.7 ^a^	0.76	0.05
Biological efficiency, DM kg/kg HCW	10.4 ^b^	10.0 ^ab^	9.60 ^a^	0.22	0.02
LM area, cm^2^	89.2	88.7	91.1	1.80	0.31
Marbling	2.38	2.57	2.40	0.09	0.21
12th-rib fat thickness, mm	5.52	5.47	5.87	0.18	0.21
Rump fat thickness, mm	8.11	8.26	8.24	0.27	0.87
Meat quality					
pH	5.92 ^b^	5.92 ^b^	5.83 ^a^	0.01	<0.01
Shear force, N	65.9	64.6	65.1	3.11	0.95
Cooking loss, %	27.4	28.0	27.5	0.50	0.69
Chroma L* (24 h)	39.6	39.7	38.9	0.85	0.77
Chroma a* (24 h)	21.6	21.2	21.2	0.54	0.84
Chroma b* (24 h)	14.8	14.9	14.2	0.51	0.57
Chroma L* (72 h)	42.7	41.8	41.9	0.92	0.73
Chroma a* (72 h)	22.7	22.0	22.4	0.63	0.76
Chroma b* (72 h)	17.0	16.6	16.6	0.61	0.85
Myoglobin, mg/g	4.22	4.20	4.15	0.04	0.35
Total lipids, %	1.72	1.90	1.76	0.09	0.12
Lipid oxidation, mgMDA/kg meat	1.05	1.03	1.05	0.01	0.15

Abbreviations: SEM—standard error of the mean; d—day; DM—dry matter; LM—*Longissimus* muscle; HCW—hot carcass weight; N—Newton; and MDA—malonaldehyde. ^1^ CTL: monensin sodium (Rumensin 200^®^; Elanco Ltd., São Paulo, Brazil) at 26 mg/kg DM. ^2^ M3NOP: monensin sodium at 26 mg/kg DM and 3-nitrooxypropanol (Bovaer^®^; DSM-Firmenich Nutritional Products Ltd., Kaiseraugst, Switzerland) at 100 mg/kg DM. ^3^ Combo: 3-nitrooxypropanol at 100 mg/kg DM; essential oils (Crina^®^ Ruminants; DSM-Firmenich Nutritional Products Ltd., São Paulo, Brazil) at 100 mg/kg DM; 25-Hydroxy-Vitamin-D3 (Hy-D^®^; DSM-Firmenich Nutritional Products Ltd., São Paulo, Brazil) at 0.10 mg/kg DM (corresponding to 4000 IU vitamin D3/kg DM); carbo-amino-phospho-chelated chromium at 4 mg/kg DM (Tortuga minerals^®^; DSM-Firmenich Nutritional Products Ltd., São Paulo, Brazil); and carbo-amino-phospho-chelated zinc at 60 mg/kg DM (Tortuga minerals^®^; DSM-Firmenich Nutritional Products Ltd., São Paulo, Brazil). Superscripts ^a, b^ signify a difference with *p* ≤ 0.05, as determined by the Tukey test.

**Table 3:** The effect of combining 3-nitrooxypropanol with different feed additives on growth performance and carcass traits of feedlot Nellore bulls fed a high-concentrate finishing diet in Exp. 2.

Item	CTL ^1^	M3NOP ^2^	Combo ^3^	SEM	*p*-Value
Growth performance					
Initial bodyweight, kg	410	410	410	3.23	0.93
d 28 dry matter intake, kg/d	11.0 ^b^	11.3 ^ab^	11.8 ^a^	0.20	0.04
d 28 average daily gain, kg/d	1.500	1.536	1.750	0.10	0.21
d 28 feed conversion, kg/kg	8.30	7.71	7.61	0.50	0.58
d 28 feed efficiency, kg/kg	0.135	0.138	0.149	0.008	0.45
d 28 bodyweight, kg	452	453	459	2.77	0.19
d 102 dry matter intake, kg/d	11.8 ^b^	11.7 ^b^	12.8 ^a^	0.23	<0.01
d 102 average daily gain, kg/d	1.814	1.765	1.892	0.05	0.36
d 102 feed conversion, kg/kg	6.57	6.61	6.78	0.16	0.61
d 102 feed efficiency, kg/kg	0.153	0.152	0.148	0.004	0.61
Final bodyweight, kg	595	590	603	5.99	0.35
Carcass traits					
Initial hot carcass weight, kg	212	213	213	3.38	0.93
Final hot carcass weight, kg	333 ^b^	331 ^b^	346 ^a^	3.89	0.04
Carcass average daily gain, kg/d	1.186 ^b^	1.157 ^b^	1.304 ^a^	0.04	0.04
Dressing, %	56.1	56.0	57.3	0.47	0.23
Carcass yield, %	65.2	65.3	68.9	1.63	0.34
Biological efficiency, DM kg/kg HCW	10.1	10.2	9.93	0.34	0.85
LM area, cm^2^	88.2	86.6	88.8	2.58	0.82
Marbling	2.35	2.41	1.93	0.17	0.11
12th-rib fat thickness, mm	5.37	6.01	5.62	0.28	0.24
Rump fat thickness, mm	7.67	8.41	7.88	0.34	0.30

Abbreviations: SEM—standard error of the mean; d—day; DM—dry matter; LM—*Longissimus* muscle; and HCW—hot carcass weight. ^1^ CTL: monensin sodium (Rumensin 200^®^; Elanco Ltd., São Paulo, Brazil) at 26 mg/kg DM. ^2^ M3NOP: monensin sodium at 26 mg/kg DM and 3-nitrooxypropanol (Bovaer^®^; DSM-Firmenich Nutritional Products Ltd., Kaiseraugst, Switzerland) at 100 mg/kg DM. ^3^ Combo: 3-nitrooxypropanol at 100 mg/kg DM; essential oils (Crina^®^ Ruminants; DSM-Firmenich Nutritional Products Ltd., São Paulo, Brazil) at 100 mg/kg DM; 25-Hydroxy-Vitamin-D3 (Hy-D^®^; DSM-Firmenich Nutritional Products Ltd., São Paulo, Brazil) at 0.10 mg/kg DM (corresponding to 4000 IU vitamin D3/kg DM); carbo-amino-phospho-chelated chromium at 4 mg/kg DM (Tortuga minerals^®^; DSM-Firmenich Nutritional Products Ltd., São Paulo, Brazil); and carbo-amino-phospho-chelated zinc at 60 mg/kg DM (Tortuga minerals^®^; DSM-Firmenich Nutritional Products Ltd., São Paulo, Brazil). Superscripts ^a, b^ signify a difference with *p* ≤ 0.05, as determined by the Tukey test.

**Table 4:** The effect of combining 3-nitrooxypropanol with different feed additives on greenhouse gas emissions of feedlot Nellore bulls fed a high-concentrate finishing diet in Exp. 2.

Item	CTL ^1^	M3NOP ^2^	Combo ^3^	SEM	*p*-Value
GreenFeed unit					
Drop, total/d	16.1	15.4	14.8	1.62	0.74
Time, minutes/visit	5.10	5.06	5.09	0.07	0.60
Drop, kg	0.56	0.54	0.52	0.06	0.54
CH_4_ production, g/d	141 ^a^	87.0 ^b^	85.6 ^b^	6.67	<0.01
H_2,_ g/d	1.23 ^b^	4.59 ^a^	5.04 ^a^	0.22	<0.01
CH_4_ yield, g/kg DMI	12.0 ^a^	7.45 ^b^	6.69 ^b^	0.50	<0.01
CH_4_ intensity, g/kg Carcass ADG	119 ^a^	75.2 ^b^	65.6 ^b^	5.43	<0.01

Abbreviations: ADG—average daily gain; SEM—standard error of the mean; d—day; DMI—dry matter intake; H—hydrogen; and CH_4_—methane. ^1^ CTL: monensin sodium (Rumensin 200^®^; Elanco Ltd., São Paulo, Brazil) at 26 mg/kg DM. ^2^ M3NOP: monensin sodium at 26 mg/kg DM and 3-nitrooxypropanol (Bovaer^®^; DSM-Firmenich Nutritional Products Ltd., Kaiseraugst, Switzerland) at 100 mg/kg DM. ^3^ Combo: 3-nitrooxypropanol at 100 mg/kg DM; essential oils (Crina^®^ Ruminants; DSM-Firmenich Nutritional Products Ltd., São Paulo, Brazil) at 100 mg/kg DM; 25-Hydroxy-Vitamin-D3 (Hy-D^®^; DSM-Firmenich Nutritional Products Ltd., São Paulo, Brazil) at 0.10 mg/kg DM (corresponding to 4000 IU vitamin D3/kg DM); carbo-amino-phospho-chelates chromium at 4 mg/kg DM (Tortuga minerals^®^; DSM-Firmenich Nutritional Products Ltd., São Paulo, Brazil); and carbo-amino-phospho-chelated zinc at 60 mg/kg DM (Tortuga minerals^®^; DSM-Firmenich Nutritional Products Ltd., São Paulo, Brazil). Superscripts ^a, b^ signify a difference with *p* ≤ 0.05, as determined by the Tukey test.

**Table 5:** The effect of combining 3-nitrooxypropanol with different feed additives on blood parameters of feedlot Nellore bulls fed a high-concentrate finishing diet in Exp. 2.

Item	CTL ^1^	M3NOP ^2^	Combo ^3^	CTL	M3NOP	Combo	CTL	M3NOP	Combo	SEM	*p*-Value		
	d 0			d 28			d 102				T	Time	T × Time
Blood parameters,													
Insulin, uIU/mL	7.18	6.68	6.99	10.3 ^a^	11.2 ^a^	9.28 ^b^	16.0 ^a^	16.1 ^a^	13.9 ^b^	0.20	<0.01	<0.01	<0.01
IGF-1, ng/mL	313	331	339	427	412	437	450	458	455	15.7	0.59	<0.01	0.78
Glucose, mg/dL	75.9	80.4	83.1	99.5 ^a^	103 ^a^	89.2 ^b^	113 ^a^	107 ^ab^	95.8 ^b^	3.72	0.05	<0.01	0.01
Albumin, g/dL	3.06	3.18	3.34	3.6	3.55	3.35	3.8	3.85	3.67	0.08	0.51	<0.01	0.01
Total protein, g/dL	6.79	6.79	6.80	6.60	6.69	6.63	6.60	6.90	6.79	0.07	0.09	0.01	0.48
Creatinine, mg/dL	1.75	1.75	1.73	1.5	1.54	1.56	2.00	2.04	2.06	0.05	0.67	<0.01	0.93
AST, u/L	68.8	67.3	69.6	78.0	80.0	64.6	82.0	93.0	79.2	4.05	0.04	<0.01	0.11
GGT, u/L	18.8	19.1	19.6	24.0	28.5	28.6	32.0	31.1	28.0	1.86	0.65	<0.01	0.19
Cholesterol, mg/dL	86.7	87.3	86.3	126	132	107	165	175	145	6.04	<0.01	<0.01	0.17
Triglycerides, mg/dL	46.3	41.7	41.6	43.0	44.7	42.1	46.0	46.2	46.5	2.21	0.64	0.15	0.59
Urea, mg/dL	48.0	48.2	46.6	46.0	47.7	47.1	36.0	39.3	41.6	1.54	0.28	<0.01	0.31

Abbreviations: SEM—standard error of the mean; d—day; DM—dry matter; T—treatments; AST—aspartate aminotransferase; GGT—gamma-glutamyl transferase; and IGF-1—Insulin-like Growth Factor 1. ^1^ CTL: monensin sodium (Rumensin 200^®^; Elanco Ltd., São Paulo, Brazil) at 26 mg/kg DM. ^2^ M3NOP: monensin sodium at 26 mg/kg DM and 3-nitrooxypropanol (Bovaer^®^; DSM-Firmenich Nutritional Products Ltd., Kaiseraugst, Switzerland) at 100 mg/kg DM. ^3^ Combo: 3-nitrooxypropanol at 100 mg/kg DM; essential oils (Crina^®^ Ruminants; DSM-Firmenich Nutritional Products Ltd., São Paulo, Brazil) at 100 mg/kg DM; 25-Hydroxy-Vitamin-D3 (Hy-D^®^; DSM-Firmenich Nutritional Products Ltd., São Paulo, Brazil) at 0.10 mg/kg DM (corresponding to 4000 IU vitamin D3/kg DM); carbo-amino-phospho-chelated chromium at 4 mg/kg DM (Tortuga minerals^®^; DSM-Firmenich Nutritional Products Ltd., São Paulo, Brazil); and carbo-amino-phospho-chelated zinc at 60 mg/kg DM (Tortuga minerals^®^; DSM-Firmenich Nutritional Products Ltd., São Paulo, Brazil). Superscripts ^a, b^ signify a difference with *p* ≤ 0.05, as determined by the Tukey test.

**Table 6:** The effect of combining 3-nitrooxypropanol with different feed additives on nutrient intake and digestibility of feedlot Nellore bulls fed a high-concentrate finishing diet in Exp. 2.

Item	CTL ^1^	M3NOP ^2^	Combo ^3^	CTL	M3NOP	Combo	CTL	M3NOP	Combo	SEM	*p*-Value		
	d 28			d 56			d 84				T	Time	T × Time
Intake, kg/d													
Dry matter	11.6	11.7	12.4	11.9	12.0	13.1	11.7	11.5	12.5	0.47	0.03	0.46	0.98
Organic matter	11.0	11.1	11.8	11.3	11.4	12.4	11.1	10.9	11.9	0.47	0.03	0.49	0.98
Crude protein	1.71	1.73	1.83	1.77	1.77	1.93	1.74	1.70	1.85	0.07	0.02	0.42	0.97
Ether extract	0.58	0.59	0.63	0.65	0.65	0.70	0.59	0.57	0.59	0.03	0.18	<0.01	0.85
Acid detergent fiber	2.17	2.21	2.30	2.13	2.16	2.37	2.34	2.30	2.52	0.09	0.02	0.02	0.96
Neutral detergent fiber	3.07	3.08	3.24	3.09	3.15	3.40	3.13	3.05	3.32	0.12	0.04	0.70	0.96
Starch	6.07	6.12	6.45	6.24	6.26	6.80	6.01	5.88	6.38	0.24	0.05	0.26	0.99
Digestibility, g/kg dry matter													
Dry matter	765	770	763	749	747	753	732	749	756	5.24	0.08	<0.01	0.07
Organic matter	779	787	778	775	769	775	756	771	772	4.68	0.25	<0.01	0.09
Crude protein	783	785	793	778	769	779	769	782	790	5.59	0.05	0.04	0.29
Ether extract	748	749	762	738	712	766	698	698	740	14.43	0.01	<0.01	0.61
Acid detergent fiber	435	470	435	390	369	411	437	441	480	16.30	0.28	<0.01	0.13
Neutral detergent fiber	579	572	582	569 ^ab^	544 ^b^	589 ^a^	559 ^b^	571 ^ab^	593 ^a^	8.04	<0.01	0.29	0.04
Starch	932	934	933	910	913	923	906	908	920	4.06	0.03	<0.01	0.48

Abbreviations: SEM—standard error of the mean; d—day; DM—dry matter; and T—treatments. ^1^ CTL: monensin sodium (Rumensin 200^®^; Elanco Ltd., São Paulo, Brazil) at 26 mg/kg DM. ^2^ M3NOP: monensin sodium at 26 mg/kg DM and 3-nitrooxypropanol (Bovaer^®^; DSM-Firmenich Nutritional Products Ltd., Kaiseraugst, Switzerland) at 100 mg/kg DM. ^3^ Combo: 3-nitrooxypropanol at 100 mg/kg DM; essential oils (Crina^®^ Ruminants; DSM-Firmenich Nutritional Products Ltd., São Paulo, Brazil) at 100 mg/kg DM; 25-Hydroxy-Vitamin-D3 (Hy-D^®^; DSM-Firmenich Nutritional Products Ltd., São Paulo, Brazil) at 0.10 mg/kg DM (corresponding to 4000 IU vitamin D3/kg DM); carbo-amino-phospho-chelated chromium at 4 mg/kg DM (Tortuga minerals^®^; DSM-Firmenich Nutritional Products Ltd., São Paulo, Brazil); and carbo-amino-phospho-chelated zinc at 60 mg/kg DM (Tortuga minerals^®^; DSM-Firmenich Nutritional Products Ltd., São Paulo, Brazil). Superscripts ^a, b^ signify a difference with *p* ≤ 0.05, as determined by the Tukey test.

## Data Availability

All data supporting the reported results are available and can be requested directly from the corresponding author.
